# *GSDMB/ORMDL3* Rare/Common Variants Are Associated with Inhaled Corticosteroid Response among Children with Asthma

**DOI:** 10.3390/genes15040420

**Published:** 2024-03-28

**Authors:** Kirsten Voorhies, Akram Mohammed, Lokesh Chinthala, Sek Won Kong, In-Hee Lee, Alvin T. Kho, Michael McGeachie, Kenneth D. Mandl, Benjamin Raby, Melanie Hayes, Robert L. Davis, Ann Chen Wu, Sharon M. Lutz

**Affiliations:** 1 Department of Population Medicine, Harvard Pilgrim Health Care Institute, Boston, MA 02215, USA; 2 Center in Biomedical Informatics, University of Tennessee Health Science Center, Memphis, TN 38163, USA; 3 Computational Health Informatics Program, Boston Children’s Hospital, Boston, MA 02115, USA; 4 Channing Division for Network Medicine, Brigham and Women’s Hospital, Boston, MA 02115, USA; 5 Division of Pulmonary Medicine, Boston Children’s Hospital, Boston, MA 02115, USA; 6 Department of Biostatistics, Harvard T.H. Chan School of Public Health, Boston, MA 02115, USA

**Keywords:** *GSDMB/ORMDL3*, asthma, inhaled corticosteroid, children, biobank

## Abstract

Inhaled corticosteroids (ICS) are efficacious in the treatment of asthma, which affects more than 300 million people in the world. While genome-wide association studies have identified genes involved in differential treatment responses to ICS in asthma, few studies have evaluated the effects of combined rare and common variants on ICS response among children with asthma. Among children with asthma treated with ICS with whole exome sequencing (WES) data in the PrecisionLink Biobank (91 White and 20 Black children), we examined the effect and contribution of rare and common variants with hospitalizations or emergency department visits. For 12 regions previously associated with asthma and ICS response (*DPP10*, *FBXL7*, *NDFIP1*, *TBXT*, *GLCCI1*, *HDAC9*, *TBXAS1, STAT6*, *GSDMB/ORMDL3*, *CRHR1*, *GNGT2*, *FCER2*), we used the combined sum test for the sequence kernel association test (SKAT) adjusting for age, sex, and BMI and stratified by race. Validation was conducted in the Biorepository and Integrative Genomics (BIG) Initiative (83 White and 134 Black children). Using a Bonferroni threshold for the 12 regions tested (i.e., 0.05/12 = 0.004), *GSDMB/ORMDL3* was significantly associated with ICS response for the combined effect of rare and common variants (*p*-value = 0.003) among White children in the PrecisionLink Biobank and replicated in the BIG Initiative (*p*-value = 0.02). Using WES data, the combined effect of rare and common variants for *GSDMB/ORMDL3* was associated with ICS response among asthmatic children in the PrecisionLink Biobank and replicated in the BIG Initiative. This proof-of-concept study demonstrates the power of biobanks of pediatric real-life populations in asthma genomic investigations.

## 1. Introduction

Asthma is the most common chronic illness in children, and inhaled corticosteroids (ICS) are the most commonly used controller medication for asthma [1]. Individual response profiles to ICS show significant and repeatable heterogeneity. For patients receiving ICS for 8–12 weeks, 35–40% of these patients do not show significant improvement in lung function [2,3]. Among asthmatic patients on maintenance ICS treatment, approximately 10% may remain at high risk of asthma attacks or being symptomatic [4]. In addition to poor adherence to medication, other important factors influencing treatment response in patients with asthma also include environmental exposures, misdiagnosis, and genetic variation [5]. 

Previously identified single nucleotide polymorphisms (SNPs) in genes, including *FCεR2*, *GLCCI1*, *CRHR1*, *T* gene, *DPP10*, *HDAC9*, *TBXAS1*, *FBXL7*, *GNGT2*, *NDFIP1*, *STAT6*, and *GSDMB*/*ORMDL3*, demonstrated association with ICS response [6,7,8,9,10,11,12,13,14]. While previous studies have found associations with common variants and ICS response for these 12 regions, no significant associations for rare variants were previously found [15,16] and examining the combined effect of rare and common variants has not been explored. Nevertheless, few studies have evaluated the potential effects of combined rare and common variants and the contribution of rare versus common variants.

To date, most pharmacogenetic studies on children with asthma have been clinical trials. However, studies in real-life populations are complementary to clinical trial studies as real-life populations account for external patient, provider, and system level factors that may moderate an intervention’s effect. The objective of this study is to: (1) assess the potential effects of combined rare and common variants and (2) conduct a proof-of-concept pharmacogenetic study in two real-world pediatric biobanks through the Genomic Information Commons [17], a collaborative across nine children’s hospitals. As a result, we examined rare and common variants associated with ICS response for 12 regions previously associated with asthma and ICS response (*DPP10*, *FBXL7*, *NDFIP1*, *TBXT*, *GLCCI1*, *HDAC9*, *TBXAS1*, *STAT6*, *GSDMB*/*ORMDL3*, *CRHR1*, *GNGT2*, *FCER2*) in the PrecisionLink Biobank with replication in the Biorepository and Integrative Genomics (BIG) initiative.

## 2. Materials and Methods

### 2.1. Discovery Population: PrecisionLink Biobank

The discovery population was at Boston Children’s Hospital PrecisionLink Biobank for Health Discovery [18], which is a site in the Genomic Information Commons [17]. The PrecisionLink Biobank links residual specimens produced as by-products of routine care to phenotypic data derived from electronic health records. Inclusion criteria included age ≥ 6 years, a diagnosis of asthma, and use of one of the following ICSs within the past year: beclomethasone, budesonide, ciclesonide, fluticasone, mometasone. For this proof-of-concept study, 91 White and 20 Black children were identified through the PrecisionLink Portal as meeting inclusion criteria and confirmed based on manual review of the electronic health record (EHR) [19]. The main outcome measure, ICS response, was defined as number of emergency department visits or hospitalizations for asthma over a one-year period based on EHRs.

### 2.2. Validation Population: BIG Initiative

The validation population was the BIG Initiative, a collaborative effort between the University of Tennessee Health Science Center and Le Bonheur Children’s Hospital [20]. Inclusion criteria for the validation population were the same as for the discovery population. In the BIG Initiative, 83 White and 134 Black children met inclusion criteria and had available WES data.

### 2.3. WES Processing for PrecisionLink Biobank Samples

DNA was obtained from the PrecisionLink Biobank. The Precision Link Biobank initiative is approved by the BCH Institutional Review Board (protocol number—P00000159). Genomic DNA (gDNA) samples were extracted from whole blood specimens of 200 subjects, using Gentra Purgene Extraction Kit or Chemagic B5k Extraction Kits and quality controlled using Quant-It assay. Gentra Purgene is part of QUIAGEN. The Boston Children’s Biobank uses a standard operating procedure to calculate and perform normalization. Afterwards, gDNA samples were shipped to the Quest Diagnostics Sequencing Center for whole exome sequencing (WES). After assessment of DNA quantity, qualified genomic DNA sample is enzymatically fragmented. Sequencing library was prepared by ligating sequencing adapters to both ends of DNA fragments. Sequencing libraries were size-selected with bead-based method to ensure optimal template size and amplified by polymerase chain reaction (PCR). Regions of interest (exons and intronic targets) were targeted using hybridization-based target capture methods (Integrated DNA Technologies xGen Exome). The quality of the completed sequencing library was controlled by ensuring the correct template size and quantity and to eliminate the presence of leftover primers and adapter-adapter dimers. Ready sequencing libraries that passed the quality control were sequenced using the Illumina’s sequencing-by-synthesis method using paired-end sequencing (150 by 150 bases). Sequenced reads were processed using GPU-accelerated genomics software suite NVIDIA ClaraTM Parabricks (v3.5.0, https://www.nvidia.com/en-us/clara/genomics/ (accessed on 10 November 2022)). We used the modules ‘fq2bam’, ‘applybqsr’, and ‘haplotypecaller’ which implements mapping reads using BWA-MEM, marking duplicated reads, recalibrating base quality scores using GATK BQSR (Base Quality Score Recalibration), and variant calling using GATK HaplotypeCaller. The Parabricks v3.5.0 is based on BWA version 0.7.15 and GATK version 4.1.0.0. The outcomes from the Parabricks suite were per-sample genomic VCF (gVCF) files, which were then used for joint genotyping using GATK4 HaplotypeCaller (version 4.2.0.0).

### 2.4. Statistical Approach

We used the combined sum test for the sequence kernel association test (SKAT) to examine the combined effect of rare and common variants on ICS response [21]. In addition, we considered rare and common variant associations separately. We used a MAF cutoff to define rare vs. common variants of 1/$\sqrt{2*Sample\;Size}$. This is the default value for the SKAT_CommonRare function that implements the combined sum test for SKAT in R. This resulted in a MAF threshold cutoff to define rare vs. common variants of 0.07 and 0.16 for the White and Black children in the PresionLink biobank, respectively and 0.08 and 0.06 for the White and Black children in the BIG Initiative biobank, respectively. The supplement contains the rs numbers for SNPs included in the analyses and classification of whether the SNP was included as a rare or common variant and whether the SNP is an intronic variant. We defined the outcome of non-response based on hospitalization or ED visit. We adjusted for age, sex, and BMI and stratified by race. Using a Bonferroni correction for the 12 regions, we used a significance threshold of 0.05/12 = 0.004. For LD pruning, we considered a shifting window of 50 SNPs, calculated the LD between each pair of SNPs in the window and removed one of a pair of SNPs if the R squared was greater than 0.5.

In the secondary analysis, for the region (*GSDMB/ORMDL3*) that was significantly associated with ICS response for the combined effect of both rare and common variants among White children in the PrecisionLink Biobank, we also analyzed the association of each SNP in the region with ICS response separately in order to examine the contribution of each SNP. Using logistic regression models, we adjusted for age, sex, and BMI. We restricted the analysis to SNPs with at least 4 variants present in order to assure convergence of the logistic regression models.

## 3. Results

For the PrecisionLink and BIG Initiative biobanks, baseline demographics are shown in Table 1 and results are shown in Table 2. Among White children in the PrecisionLink Biobank, *GSDMB/ORMDL3* was significantly associated with ICS response for the combined effect of both rare and common variants (*p*-value = 0.003). This signal replicated in the BIG Initiative (*p*-value = 0.02). When we stratified by rare and common variants in the *GSDMB/ORMDL3* region, both rare (*p*-value = 0.008) and common variants (*p*-value = 0.03) were marginally associated with ICS response among White children in the PrecisionLink Biobank. However, common variants (*p*-value = 0.03) and not rare variants (*p*-value = 0.30) were marginally associated with ICS response in the BIG Initiative. In addition, *STAT6* was associated with ICS response for the combined effect of rare and common variants (*p*-value = 0.003) among Black children in the PrecisionLink Biobank. However, the sample size included only 20 children and the signal did not replicate in the BIG initiative.

For the secondary analysis, the results are displayed in Table 3 for the association of ICS response with the individual SNPs in *GSDMB/ORMDL3* among White children in the PrecisionLink Biobank for SNPs, with at least four variants present. While the joint effect of the common variants (*p* = 0.03) and rare variants (0.008) were separately associated with ICS response in Table 2, there is not a single common variant that is strongly associated with ICS response as seen in Table 3. The joint association of the common variants in *GSDMB/ORMDL3* with ICS response seems to be due to several SNPs in the region and not the effect of a single SNP. Also, note that all of the SNPs that were classified as common variants in *GSDMB/ORMDL3* are also intronic variants.

## 4. Discussion

Using WES data, *GSDMB/ORMDL3* was significantly associated with ICS response for the combined effect of rare and common variants (*p*-value = 0.003) among White children in the PrecisionLink Biobank and replicated in the BIG Initiative (*p*-value = 0.02). *GSDMB/ORMDL3* has previously been associated with ICS response and exacerbations for common variants [22], asthma for common variants [23], and asthma for the combined effect of rare and common variants [24]. However, *GSDMB/ORMDL3* has not previously been associated with ICS response or exacerbations for rare variants. While the association of asthma exacerbations or ICS response with rare variants has been studied previously, no significant associations were previously found [15,16].

While the objectives of this study were to assess the association of combined rare and common variants on ICS response and to conduct a proof-of-concept pharmacogenetic study in two real-world pediatric biobanks, there were limitations for this study. The sample sizes were small and evaluation in larger cohorts is needed. While *STAT6* was significantly associated with ICS response among Black children in the PrecisionLink Biobank, caution is needed for this result since the sample size is extremely small and the signal did not replicate. Also, while the outcome was based on EHRs for Boston Children’s Hospital, outpatient, ED visits, and hospitalizations that occurred at other sites may have been missed.

Future studies are needed due to the small sample size for this study. Given the complexity of asthma and its treatment responses, larger and more diverse samples could potentially provide a more robust understanding of the genetic determinants involved. Future studies incorporating preliminary evidence from functional or structural predictions regarding the identified variants could further strengthen this study’s conclusions. The clinical relevance of these findings would be enhanced by such evidence that could offer insights into the biological mechanisms through which these variants influence ICS response.

Large biobanks that link collections of biological specimens to clinical information in diverse, real-life populations have been fruitful in the discovery of pharmacogenetic markers. Nevertheless, few such biobanks are dedicated to children, likely because of logistical and regulatory issues related to the recruitment of children. Nevertheless, including children is important to ensure that children benefit from genetic advancements. This proof-of-concept study demonstrates the potential power of biobanks of pediatric real-life populations in genomic investigations for asthma.

## Figures and Tables

**Table 1:** Characteristics of participants for the PrecisionLink Biobank and Biorepository and Integrative Genomics (BIG) Initiative.

	PrecisionLink Biobank		BIG Initiative	
	White (N = 91)	Black (N = 20)	White (N = 83)	Black (N = 134)
Age in years, mean (SD)	14.5 (5.8)	14.5 (5.4)	15.7 (4)	15.9 (3.8)
Sex (female), N (%)	54 (56%)	9 (45%)	33 (39.8%)	64 (47.8%)
BMI, mean (SD)	23.5 (7.1)	28.9 (11.3)	25.0 (7.9)	29.1 (11.2)
Experienced asthma exacerbation, N (%)	14 (15%)	9 (45%)	52 (62.7%)	94 (70.1%)

**Table 2:** Results of the combined common and rare variant analyses. Analyses are also stratified by rare and common variants giving the *p*-value for the combined sum test and the number of SNPs included in the analysis. Bonferroni significant results are highlighted in green and marginal associations (*p*-value < 0.05) are highlighted in yellow. Note that the column “BP” for base pairs refers to genome build GRCh38/hg38, and note that the columns “# SNP” is the number of SNPs.

			PrecisionLink Biobank (White)						PrecisionLink Biobank (Black)						BIG (White)						BIG (Black)					
			Common + Rare		Common		Rare		Common + Rare		Common		Rare		Common + Rare		Common		Rare		Common + Rare		Common		Rare	
Chr	Gene	BP	P	# SNP	P	# SNP	P	# SNP	P	# SNP	P	# SNP	P	# SNP	P	# SNP	P	# SNP	P	# SNP	P	# SNP	P	# SNP	P	# SNP
2	*DPP10*	114,442,299–115,845,780	0.23	108	0.39	18	0.11	90	0.39	80	0.62	8	0.14	72	0.85	38	0.72	9	0.78	29	0.17	85	0.20	19	0.39	66
5	*FBXL7*	15,500,180–15,939,795	0.12	22	0.21	1	0.03	21	0.07	7	-	0	0.07	7	0.91	6	0.74	1	0.95	5	0.11	20	0.13	4	0.04	16
5	*NDFIP1*	142,108,779–142,154,440	0.76	23	0.74	6	0.17	17	0.92	19	1	1	0.44	18	0.36	1	-	0	0.36	1	0.77	19	-	0	0.77	19
6	*TBXT*	166,157,656–166,168,700	0.44	33	0.13	17	0.45	16	0.1	30	0.08	10	0.05	20	0.76	17	0.52	8	0.15	9	0.72	27	0.66	13	0.44	14
7	*GLCCI1*	7,968,796–8,094,272	0.10	33	0.03	9	0.65	24	0.11	29	0.03	7	0.07	22	0.52	13	0.50	6	0.45	7	0.43	27	0.61	7	0.33	20
7	*HDAC9*	18,086,825–19,002,416	0.42	80	0.21	14	0.75	66	0.05	75	0.11	14	0.26	61	0.76	30	0.99	8	0.06	22	0.46	79	0.27	21	0.69	58
7	*TBXAS1*	139,777,051–140,020,325	0.99	73	0.99	16	0.62	57	0.45	51	0.34	13	0.23	38	0.61	29	0.50	4	0.60	25	0.04	52	0.03	8	0.10	44
12	*STAT6*	57,095,408–57,132,139	0.94	32	0.88	8	0.62	24	0.003	35	0.003	3	0.05	32	0.33	16	0.66	3	0.07	13	0.23	36	0.63	5	0.21	31
17	*GSDMB*/*ORMDL3*	39,904,595–39,927,601	0.003	36	0.03	12	0.008	24	0.39	36	0.18	7	0.22	29	0.02	13	0.03	4	0.30	9	0.57	26	0.52	5	0.65	21
17	*CRHR1*	45,784,277–45,835,828	0.23	30	0.35	10	0.55	20	0.14	24	0.11	3	0.18	21	0.32	13	0.24	6	0.24	7	0.71	41	0.41	5	0.89	36
17	*GNGT2*	49,202,791–49,210,574	0.07	7	0.03	3	0.3	4	0.04	7	-	0	0.04	7	0.50	5	0.31	3	1.00	2	0.43	7	0.37	3	0.49	4
19	*FCER2*	7,688,758–7,702,146	0.87	53	0.54	22	0.98	31	0.16	38	0.23	15	0.08	23	0.20	16	0.17	9	0.20	7	0.71	34	1.00	10	0.54	24

**Table 3:** Results of the single variant logistic regression analyses for *GSDMB/ORMDL3* in White children in the PrecisionLink Biobank for SNPs with at least 4 variants present.

SNP	BP	Intronic Variant	Common vs. Rare Variant	MAF	Beta	SE	*p*
rs36000226	39,907,676	Yes	Common	0.36	0.93	0.42	0.03
rs1185154183	39,907,727	Yes	Common	0.30	0.66	0.42	0.12
rs117097909	39,908,718	Yes	Common	0.09	−1.36	1.1	0.22
rs2290400	39,909,987	Yes	Common	0.43	1.07	0.48	0.02
rs870830	39,912,120	Yes	Common	0.26	−0.93	0.59	0.12
rs870829	39,912,129	Yes	Common	0.33	0.6	0.44	0.18
rs921650	39,912,823	Yes	Common	0.45	0.88	0.43	0.04
rs113894104	39,916,702	Yes	Rare	0.03	1.88	1.02	0.07
rs1035064486	39,917,588	Yes	Common	0.45	−0.45	0.37	0.23
rs111379006	39,918,847	No	Rare	0.02	1.61	1.34	0.23
rs4065275	39,924,612	Yes	Common	0.47	1.33	0.53	0.01
rs8076131	39,924,659	Yes	Common	0.48	0.6	0.4	0.13
rs12603332	39,926,554	Yes	Common	0.44	1.02	0.49	0.04
rs17608925	39,926,578	Yes	Common	0.09	1.42	0.71	0.05

## Data Availability

The data are not currently publicly available but will be publicly available in the future or potentially accessible through a biobank collaborator.
